# A Qualitative Study Exploring the Experiences and Perceptions of Patients with Hemophilia Regarding Their Health-Related Well-Being, in Salamanca

**DOI:** 10.3390/jcm12165417

**Published:** 2023-08-21

**Authors:** Laura Ramos-Petersen, Juan Antonio Rodríguez-Sánchez, Jonathan Cortés-Martín, Andrés Reinoso-Cobo, Juan Carlos Sánchez-García, Raquel Rodríguez-Blanque, Juan R. Coca

**Affiliations:** 1 Department of Nursing and Podiatry, Faculty of Health Sciences, University of Malaga, Arquitecto Francisco Peñalosa 3, Ampliación de Campus de Teatinos, 29071 Malaga, Spain; lauraramos.94@uma.es; 2 Department of Biomedical Sciences and Diagnosis, University of Salamanca, C/Alfonso X el Sabio S/N, 37007 Salamanca, Spain; jarshm@usal.es; 3 Research Group CTS1068, Andalusia Research Plan, Junta de Andalucía, Nursing Department, Faculty of Health Sciences, University of Granada, 18071 Granada, Spain; jcortesmartin@ugr.es (J.C.-M.); jsangar@ugr.es (J.C.S.-G.); rarobladoc@ugr.es (R.R.-B.); 4 San Cecilio Clinical University Hospital, 18016 Granada, Spain; 5 Social Research Unit on Health and Rare Diseases, Sociology and Social Work Department, University of Valladolid, 47002 Valladolid, Spain; juanr.coca@uva.es

**Keywords:** hemophilia, rare disease, health inequalities, social impact

## Abstract

Hemophilia is a chronic, congenital/hereditary and X-linked disease, characterized by an insufficiency of factors VIII or IX, which are necessary for blood clotting. Those affected by hemophilia often suffer from particular psychosocial problems, both in the acceptance, coping, treatment and self-management of their disease and in their family and social relationships, which are often mediated by these circumstances. The aim of this study was to explore the experiences of people with hemophilia or their family members, of in a specific region of Spain, regarding the impact of having hemophilia. Structured interviews were conducted and developed, using the studies of the World Federation of Hemophilia and Osorio-Guzmán et al. as a guide, as well as a literature review of qualitative work on hemophilia. Data were analyzed using a six-step thematic analysis. A total of 34 interviews were thematically analyzed. The results showed that three key themes emerged from the data: (1) the daily impact of having hemophilia, (2) uncertainty about the disease, (3) the role of associations and (4) support from institutions. The results make it clear that the disease has a major impact on their lives (work, family, leisure and personal environment). The main conclusion is that hemophilia has a negative impact on the daily lives of patients, families and caregivers.

## 1. Introduction

Hemophilia is an X-linked disease, associated with mutations affecting the factor VIII or factor IX gene located on the X chromosome; it is classified into type A (factor VIII) and type B (factor IX) [1]. The global prevalence of hemophilia A is about 1 in 5000 male births, and 1 in 30,000 male births for hemophilia B. These figures are similar worldwide, regardless of ancestry or ethnicity. Being linked to the X chromosome, the incidence is higher in men, and women are carriers without developing the disease [2].

Until a few decades ago, it was presented as a disease with a low life expectancy [3]; with advances in treatments, the associated mortality rate has decreased considerably [4], and currently life expectancy is estimated to be slightly below the average of the population in developed countries [5].

Hemarthrosis and bleeding at the muscular level are one of the main clinical features of hemophilia [6], causing a progressive decrease in mobility due to musculoskeletal deterioration; in severe clinical cases, osteoarticular deformation with shortening of the limbs is reported; this progressive deterioration generates an increase in disability [7].

The most commonly described joints affected in the literature are the knee and the ankle, causing balance disturbances and antalgic gait, depending on the pain and stiffness of the patient; one treatment option is osteoarticular surgery [8].

With the evolution of the disease, depending on the severity, level and frequency of hemorrhages, there is chronic pain, increased disability and deterioration of the quality of life at the physical and psychological levels [9,10,11], so it is recommended to introduce treatment in childhood in order to protect bone formation, compared to adulthood, which would minimize the damage that could be generated at the ostearticular level [12,13]. It is estimated that children and people with access to early intensive treatment means a prevention of limiting arthropathies [14]; however, there is no consensus on what methods should be used in studies to monitor patients in long-term studies [15].

Current treatment means a reduction in spontaneous bleeding [16] and promotes a change in attitude and normalization in patients’ activities of daily living. Currently, physical activity and low-impact exercise are recommended [17,18]; controlled physical activity in patients under treatment is not associated with a significant increase in the incidence of bleeding, as this will depend on the severity of the disease and adherence to treatment [19].

Pharmacological advances do not show a direct correlation with the patient’s perception of safety; fear of trauma and its potential complications [20] encourages a sedentary lifestyle [21]. The decrease in productivity due to sick leave, treatments, medical appointments or even the fear of contagion of blood-borne diseases, among others, generates social isolation and conditions the perception of the patient’s quality of life [22].

Associated with hemophilia, psychodepressive and anxiety signs have been described in 40% of patients, both in patients and their immediate family members in the case of pediatric patients, and are associated with an exacerbation of symptoms and difficulties in adherence to treatment [23]. 

In recent years, studies have been published analyzing quality of life with different standard evaluation methods in an attempt to assess the patient’s perception, but they lose sensitivity because they are not specific [24].

In view of the above, the study of the impact of the disease on the lives of patients and family members diagnosed with hemophilia is of social and clinical interest and which should be addressed, which is the justification for this report. Knowledge of the experiences and perceptions of these patients and their families will help to advance our understanding of this pathology. By knowing the repercussions on daily life and the environment caused by this disease, a scenario with a wide range of options for working to increase the quality of life of these patients by preventing the impact will be proposed. 

The aim of the qualitative study was to analyze the concerns of hemophilia patients and their families, and the repercussions on their lives and environment, taking into account the public health resources available to them. To achieve this objective, it was decided to select a specific sample of patients who shared a series of clinical criteria and, at the same time, belonged to the same region of the country so that the data obtained would be significant and extrapolable. In this case and after the sample study, the region selected was Salamanca.

## 2. Methods

The present study adopted a qualitative approach to explore the experiences of people with hemophilia in terms of the social inequalities because of their disease and their health-related well-being.

### 2.1. Participants

A convenience sample of people with hemophilia was recruited from the Spanish association of hemophilia.

Inclusion criteria were that the participant was consenting, aged 18 or over, had a diagnosis of hemophilia A or B with a family member diagnosis of hemophilia and had the ability to express themselves in Spanish to understand and respond to the questions.

Participants were excluded if they presented with another blood-clotting-related disease or present cognitive impairment.

Once the process of recruitment and screening of participants had been carried out, the sample obtained was studied quantitatively and, for reasons of sample size, it was decided to select only participants from the Salamanca region. Salamanca is a Spanish province with a total of 333.649 habitants.

Finally, eligible participants were contacted by phone to ascertain willingness to participate in the qualitative study.

They all agreed to take part in the study and provided informed and written consent. Interviews took place from October to December 2022.

### 2.2. Data Collection 

Data were collected using a structured interview. Multiple interviews were carried out to achieve data saturation, meaning that the themes are repeated in some interviews. The following data (mainly quantitative) were also collected prior to the start of the study:

- Characteristics of the participant: gender, academic training, years of age and number of family members with hemophilia.
- Characteristics of the patients with hemophilia (it could be the participants themselves or a family member): year of age, hemophilia type, previous family history of the disease and how long it was from the first symptoms to the diagnosis.

The interviews were conducted by phone, and they were recorded using a digital voice recorder. Furthermore, field notes supplemented the data. The interviews were carried out by a researcher who had experience with patients with hemophilia from both a clinical and research context, and participants were made aware that the researcher was a nurse. A code was assigned for each patient, so they were anonymized or pseudonymized. There was only one person from the research team (data manager) who knew the relationship between the assigned code and the participants’ data, leading to a blind code. That member accessed the files with a password and was not part of the data collection process, so not to interfere. 

Informed consent forms were printed and sent to be signed by the participants. All of these signed consents, together with the data collection sheets and field notes, are archived in an office for 5 years, to which only authorized members have access. After 5 years, those folders will go to a warehouse belonging to the nursing department of the University of Granada, where all the documents related to studies that have been carried out are kept, and they will remain under lock and key for 15 years. Later, they will be destroyed.

Data analyses were conducted by two researchers. The questions for the structured interview were developed from a review of the literature on hemophilia [2,25] The research question that was addressed in this qualitative study was as follows: How does hemophilia impact on the lives of diagnosed patients and their families? Therefore, the following questions were asked to the participants:

- How has your life changed since living with this pathology?
- Has the illness conditioned school or employment insertion?
- Do you consider that this disease has conditioned your social or partner relationships?
- Does your family suffer from any kind of uncertainty due to the disease?
- Do you trust your doctors?
- Do you think that doctors can be biased, which could affect you or your family?
- Do you consider the work of patient organizations useful?
- Do you think that you or your family have the best treatment for your illness?
- Do you consider that health institutions should provide more help (devices, training, specific areas, etc.) to people with hemophilia?
- Do you consider that political institutions should take more specific actions for people with hemophilia?

No repeat interviews were conducted, and transcripts were not returned to participants for comment. 

In light of the above, it should be noted that, in order to reduce the risk of bias, all documentation was treated with transparency at all times and the analysis of the data obtained was subject to peer review and audit.

### 2.3. Data Analysis

The six-step thematic analysis framework from Braun and Clarke [26] was followed for the data analysis. One researcher undertook a line-by-line analysis of the transcribed experiences of each participant. 

From that work, another researcher (LRP) read the field notes and all of the transcripts, and codes were developed. Initial codes were then collated to gather data into themes. Particular attention was paid to both the frequency of emerging codes and their importance for multiple participants. Coded extracts were reviewed within their themes and, afterward, defined and named. MAXQDA qualitative data analysis software was used to facilitate coding and analysis. The findings were then scrutinized by the co-authors. 

### 2.4. Ethical Considerations

This qualitative study received ethical approval from the committee of the CEIM/CEI Province of Granada Ethics Committee with the code 02032021. This study was carried out in full accordance with the provisions of the Declaration of Helsinki regarding ethical principles for medical research involving human subjects and was approved by the Ethics Committee.

## 3. Results

A total of 34 interviews were analyzed thematically. In total, 19 of the participants were female, and 15 were male, aged between 20 and 80 years old. 

The study sample consisted of 55.9% males and 44.1% females, of whom 55.9% had higher education; more than half (76.5%) were under 50 years of age, the study sample had a median of 2 family members with hemophilia, with 64.7% having a family history. With reference to the time of diagnosis, 58.8% were diagnosed in less than 6 months and 23.5% in more than one year.

Table 1 below shows the most relevant data from the quantitative analysis of the sample. 

A total of 115 codes were identified, which were then organized into four themes. The resulting themes were agreed by the researcher and co-authors to enhance the validity of the data:

- Daily impact of having hemophilia.
- Uncertainty about the disease.
- Role of associations.
- Support from the institutions.

### 3.1. Theme 1: Daily Impact of Having Hemophilia

When the relatives of patients with hemophilia were asked how their lives have changed since they have been living with the disease, the vast majority of them stated that many aspects of their lives are totally different, including their work, family, leisure and personal environments. In particular, they highlighted the stress and fatigue caused by the care required by this type of patient, especially if they are children.

*“They are absorbent cotton children.”*

(participant 3)

*“It has influenced the decision not to have more children.”*

(participant 31)

*“We have had to learn to manage the daily stress it causes us.”*

(participant 4)

*“It influences the way we treat ourselves as we are afraid of hurting ourselves and having bleedings.”*

(participant 19)

Some participants indicated the adaptations they have had to make in their day-to-day lives:

*“We plan our trips very carefully.”*

(participant 22)

*“You have to adapt. You have to know how to choose sports, avoiding contact sports. Life changes radically when there is a person in the family who suffers from it.”*

(participant 25)

*“He did not attend school for the first few years so that prophylaxis could be introduced gradually.”*

(participant 3)

For some of the interviewees, the disease also influences their working life, to the extent that they have had to stop working, change jobs or reduce their working day. In some cases, participants explained that the reason for this is that they do not trust someone else to take care of their family members with hemophilia, and sometimes this care means that they have to leave their job suddenly, making it impossible to combine both activities. In addition, some participants with hemophilia feel discriminated against because of the disability resulting from their disease:

*“I have reduced working hours to be able to apply the best care to my son.”*

(participant 31)

*“I stopped working and we go out little.”*

(participant 4)

*“They always question whether a person with a disability can fulfill the tasks.”*

(participant 15)

*“One comes late to work in order to take the children to the hospital.”*

(participant 25)

Regarding the social relationships of the participants, there has been a great discrepancy of opinions. Some felt that the disease has not conditioned them socially at all or that at the beginning it did, but they overcame those barriers. In contrast, others feel that they are very conditioned, for reasons such as fear, and feeling vulnerable and different from the rest.

*“People find it difficult to stay and take care of my son because they are much more afraid that he might fall or give them a blow...so we have little time for the couple, even time for oneself as all the responsibility is on me since the child is small and I am the only one in my family who knows how to administer intravenously the coagulation factor.”*

(participant 26)

*“In my case, having children would be a risk, it conditions the search for a partner.”*

(participant 28)

*“If you don’t know how to transmit confidence that you can handle what is happening to you perfectly, you generate doubts in the other person and in the end they move away.”*

(participant 29)

### 3.2. Theme 2: Uncertainty about the Disease

When participants were asked if they felt uncertainty due to the disease, half indicated that they did and half indicated that they did not. Those who did indicate feeling uncertainty referred to it mainly regarding the safety of their family members, that they would not be harmed, dependence on treatment or joint mobility:

*“Now he is in adolescence, a stage of change, we trust him, but you are always worried about going out on the weekends, until he comes back safe and sound.”*

(participant 5)

*“When he is not with me I am a bit anxious hanging on the phone.”*

(participant 6)

*“I am afraid that my children will have a traffic accident and that there is no one who can indicate that they are hemophiliacs.”*

(participant 7)

*“I am uneasy about factor dependency, if you forget or don’t have enough on a trip outside of Spain.”*

(participant 24)

*“I have uncertainty about my joints, if I will have mobility problems resulting from the bleedings.”*

(participant 28)

This uncertainty is not about the hematologist doctor per se, since the participants indicated that they trust them; however, although the vast majority do not feel that doctors have some conditioning factors that affect them, some participants think they may be influenced by the pharmaceutical industry, appointment time or economic reasons, which limits them from implanting new treatments or diagnostic tests. However, they do feel uncertainty with respect to other healthcare professionals:

*“The health centers, when it comes to administering the factor, do not know.”*

(participant 10)

*“It depends on the doctor on duty, on the experience of the healthcare personnel, on whether they are up to date with the latest innovations and treatments, on the money available in the hospital, on whether they do more or less diagnostic tests, etc.”*

(participant 26)

*“Hematologists know how to work against the disease, but the rest of the specialists do not give answers because they are hesitant and it is very sad.”*

(participant 30)

*“We need a multidisciplinary team, with a traumatologist and a physiotherapist, to prevent future interventions such as knee prostheses in young boys.”*

(participant 34)

When it came to asking the participants if they felt they had the best treatment for the disease, most feel that they have a good treatment, but not the best available. They refer to other countries where they have other treatments that are better regarded. That is, they feel they have adequate treatment, but improvable:

*“From my point of view in my family I have the best treatment that is being used in Spain; but not the best available in the market.”*

(participant 19)

### 3.3. Theme 3: Role of Associations

100% of the participants consider the work of patient associations to be useful. They feel that they help them, both emotionally and in resolving doubts, especially in cases where there is no previous history of the disease in the family. In addition, they have the function of connecting people who are in a similar situation, which makes them feel more understood. Therefore, they believe that the role of associations is fundamental in helping to overcome the disease.

*“They give voice and visibility to a collective.”*

(participant 5)

*“Thanks to them I met people with experience who had already gone through the same case.”*

(participant 26)

*“In the first moments they get you out of the initial shock of receiving the diagnosis and later you can share experiences with people who understand and empathize with you as they go through the same circumstances as you.”*

(participant 32)

Another factor that they appreciate about the associations is that they help them to stay updated:

*“They are necessary and indispensable, always aware of any novelty, progress or news regarding the disease.”*

(participant 13)

In addition, all of this adds to the fact that the participants feel that, thanks to the associations, they experience more involvement from the institutions:

*“An association works to help and improve the quality of life of its members as much as possible, as well as to defend their rights and address their concerns.”*

(participant 19)

*“Without them, when it comes to demanding rights, we would be ignored.”*

(participant 22)

*“They do a great job of raising awareness and sensitizing society.”*

(participant 27)

### 3.4. Theme 4: Support from the Institutions

When asked how healthcare institutions could improve their treatments, participants suggested more health education (both to society and to doctors), psychological support, rehabilitation, specific treatment units where they feel they are getting the best care when they go to medical centers and that all of this does not depend on where they live:

*“From the health centers they could give informative talks.”*

(participants 5)

*“We need multidisciplinary treatment, especially hematology and traumatology.”*

(participant 23)

*“They need to have a specialized unit with traumatologists specializing in hemophilia, rehabilitators, physiotherapists, nurses. Any mishap that may occur can be solved there.”*

(participant 27)

Regarding institutions in general, the participants request more help in the area of employment, such as help with job placement, job orientation and resources. Also, economic support in general, such as aid to be able to afford sports activities beneficial to the disease:

*“Financial support for parents who have to stop working to care for their hemophiliac child. In the end, we mothers are the first doctors and nurses, and it is difficult to reconcile this with our work schedule.”*

(participant 32)

*“More scholarships and places for the disabled, since they need extra support, and their limitations should be taken into account.”*

(participant 26)

*“To have more possibilities for activities according to circumstances, such as free swimming courses.”*

(participant 4)

In addition, they demand more recognition of the disability they suffer:

*“It is necessary to unify the percentages of disability, and that there were no differences between communities.”*

(participant 5)

*“We need hemophilia to be included in the list of serious diseases and for the disability to be recognized.”*

(participant 6)

They also request support for research, including new drugs or form of application.

## 4. Discussion

The aim of this study was to determine the impact and repercussions of hemophilia on patients diagnosed with hemophilia and their families. The repercussions, in terms of the development of their daily life, take into account aspects such as independence, social integration, adaptation to the work environment, access to health institutions and the relationship with the world of the associative movement. For this purpose, a series of participants with a series of common clinical characteristics and belonging to the same territorial region were selected, in order to be able to compare and relate all experiences and perceptions in a coherent manner and avoid the risk of bias.

The results obtained suggest that hemophilia has a drastic impact on all of the levels mentioned above [1]. It hinders the independent development of daily activities and hinders social and occupational integration [2]. Regarding the relationship with the health institution, some disappointment is observed in terms of the level of knowledge about the disease and the clinical approach to this type of patient and their families (www.ern-rnd.eu, accessed on 29 June 2023). On the other hand, the relationship with patient associations always presents good references [27].

Analyzing the results on the impact caused by this disease in more detail, it can be seen that there is unanimity that the repercussions exist and are of a negative nature, influencing the different sectors that are part of the vital development. The repercussions on the work environment occur when, for reasons of complications of the disease, the need for medical care, hospital admissions, administration of prophylactic treatments or simply due to occasional indisposition, the work schedule must be modified and made compatible [4]. There are studies that speak of rejection at work and stigmatization of patients diagnosed with a rare disease. Regarding the family environment, and coinciding with the studies of Lin et al. [28], levels of dependence are observed that increase as the disease progresses. It is necessary to highlight the role of the main caregiver in the family environment, who should be taken into account as an essential character in the development of the disease, but should not be forgotten as an independent patient. There are studies, such as that of Alfaro et al., focused on the care of the main caregiver, addressing the difficulties generated by being the caregiver of a patient with a rare chronic disease [29], and the repercussions at the personal level of the caregiver are also evident. A patient diagnosed with hemophilia needs to adapt to a life with the disease. He/she needs to know the clinical features and complications of the pathology and to seek solutions to the underlying problems. He must know his body’s limits and avoid risky exposures on a daily basis. The study by Stephensen et al. on the physical limits of a patient with hemophilia stands out [11].

Regarding social relationships, there is a certain dichotomy in the results obtained in this study. There is no unanimity in the perceptions of the interviewees, as some claim not to have any social repercussions and others do. Studies on psychosocial aspects in rare diseases relate this fact to the age of the patient [30].

Another aspect presented in the results of this report is the state of uncertainty to which patients diagnosed with hemophilia are exposed. This fact is closely related to the stage of diagnosis and development of the disease. The delay in diagnosis, characteristic of rare diseases in general and of this pathology in particular [31], hinders the clinical approach to the disease, plunging the patient into a situation of uncertainty and chaos that has a harmful influence on his or her state of health, aggravating the symptoms of the disease and enhancing the appearance of mental disorders. The delay in diagnosis is a major problem for this field [32]. 

The average time to diagnosis of hemophilia can vary depending on different factors, such as access to care, awareness of the disease and the availability of diagnostic tests. Overall, the median time to diagnosis of hemophilia has decreased in recent decades due to advances in awareness and increased availability of genetic testing. Today, in many developed countries, the average time to diagnosis of hemophilia can range from 1 to 4 years, although this period can vary significantly. It is important to note that some cases of hemophilia can be diagnosed at birth if there is a known family history or if neonatal screening is performed. However, in other cases, diagnosis may be delayed until childhood or adulthood, especially if the initial symptoms are mild or confused with other conditions. Delayed diagnosis of hemophilia can have negative consequences for patients, as they may experience recurrent bleeding and potentially joint damage before receiving adequate treatment. It is, therefore, important to promote greater awareness of hemophilia and ensure timely access to diagnostic testing, especially in those patients with non-specific symptoms or a family history of the disease. It is essential that patients with suspected symptoms of hemophilia are assessed by healthcare professionals with expertise in bleeding disorders and, if necessary, referred to hematology specialists. Multidisciplinary collaboration between primary care professionals, hematologists and other specialists can be crucial for early diagnosis and the appropriate management of hemophilia [33].

Depression and anxiety are the most common disorders in long-term diseases [34]. Most patients diagnosed or in the diagnostic phase of a rare disease suffer from anxiety and depression due to uncertainty regarding their health status, increasing the psychological burden and their psychopathology [30,35].

These patients’ uncertainty about their health status is sometimes closely linked to the proposed treatment. Current treatments for hemophilia focus on the replacement of deficient clotting proteins and the control of bleeding. Clotting factor replacement therapy should be highlighted, as it is presented as the main treatment for hemophilia. Concentrates of missing clotting factor (factor VIII in hemophilia A or factor IX in hemophilia B) are administered by intravenous infusion. These concentrates can be derived from human blood or produced by genetic engineering techniques. The aim is to raise clotting factor levels in the body to prevent or treat bleeding. It is true that there are a number of limitations such as the frequency of administration, as regular infusions are required, which can be inconvenient and affect the quality of life of patients. Another limitation is the development of inhibitors, which are antibodies that neutralize the administered clotting factors. This may hinder treatment efficacy and require alternative approaches. In any case, clotting factor concentrates are expensive, which may limit their accessibility for some patients and health systems [36].

Gene therapy advances in this disease are significant. There is a technique in development that seeks to correct the underlying genetic deficiency in hemophilia. A functional clotting factor gene is introduced into the patient’s cells so that they can continuously produce the missing factor. Realistically, though initial results are promising, long-term follow-up is required to assess the efficacy and safety of the gene therapy [37].

The role of patients’ associations is decisive in these cases. They form a solid network that provides support in different aspects for this type of patients. They provide up-to-date information on the disease, welcome the newly diagnosed patient and establish contacts between patients suffering from the same type of disease. At the same time, they form an important movement characterized by their selfless dedication to the advancement of this type of pathology. They offer reassurance and support patients and their families throughout the disease process. Studies such as those of the European Reference Networks highlight the value of the associative movement in this type of disease.

When talking about possible improvements in the healthcare system, a wide range of aspects stand out, such as the economic aspect, for example. Data collection shows that many patients affected by this pathology report a deficit in terms of investment in social assistance, scholarships for job placement and even in the choice of treatments, as shown in the study by Srivastava et al. [19]. 

Providing affordable treatments for hemophilia patients requires the implementation of strategies by institutions and governments. 

Governments should negotiate agreements with drug manufacturers to obtain lower prices for clotting factor products. This may include bulk purchasing, implementing compulsory licensing, or encouraging competition among manufacturers [38].

In addition, more patient-friendly health coverage policies could be put in place to ensure affordable access to hemophilia treatments. This may include the inclusion of clotting factor concentrates in public or private health insurance plans, as well as the removal of barriers to access, such as age limits or income restrictions.

Financial support programs are needed, where institutions and governments can help hemophilia patients access treatment. These programs may provide grants, scholarships or assistance in paying for medication, medical consultations and laboratory tests [39].

Education and awareness raising are essential in these cases. It is important for institutions and governments to promote hemophilia education and awareness. This can help remove the stigma associated with the disease and promote early detection, which, in turn, can lead to more effective and less costly treatment.

It is essential that strategies are tailored to the specific needs and resources of each country or region. Collaboration between governments, drug manufacturers, healthcare professionals and patient organizations is also crucial to effectively address affordable access to hemophilia treatment [38].

The management of hemophilia can vary between countries due to factors such as access to medical resources, health policies, availability of treatments, and differences in healthcare infrastructure. Access to treatment, health insurance coverage, healthcare infrastructures and the level of education and awareness of the disease are aspects that will mark the main differences in the management and management of hemophilia between countries.

Another aspect that will undoubtedly have an impact on the management of hemophilia is the health policies and approaches to hemophilia management in each country’s health system. Some countries may have specific policies for the management of hemophilia, while in others, the approach may depend more on the initiative of individual health professionals. It is important to note that these differences are not exhaustive and that the approach to hemophilia may vary even within countries due to regional and cultural factors, as is the case in the sample selected for this study. Salamanca belongs to the Castilla y León Health System (better known by its acronym Sacyl), and health policies may be different from those of, for example, Granada, which is a region belonging to the Andalusian Health System, although both regions are part of the Spanish national health system. Importantly, collaboration between countries and the dissemination of best practices can contribute to improving the approach and management of hemophilia worldwide [19].

Another aspect for improvement is that the level of knowledge of health professionals about hemophilia is low and sometimes limiting when it comes to the clinical approach, as observed in the study by Chaigneau et al. [40].

It is also necessary to highlight the need for research projects to be concerned with the progress of this pathology, as Kennedy et al. did [17].

Most of the patients interviewed proposed improvements to the current healthcare system, with some of them coinciding with those presented in the study by Lopez-Cortacans et al., in their proposal to improve the healthcare system [41].

An important aspect that should be mentioned in this report and that cuts across all of the fields discussed above, as it is closely related to quality of life, is motivation. Motivating a hemophilia patient can be a challenge, as living with a chronic disease can be emotionally draining and physically limiting. However, there are some strategies that can help motivate hemophilia patients [42]:

- Education and understanding: providing the patient with a thorough understanding of their disease and its treatment. Explaining how hemophilia works in their body, how they can control bleeding and how appropriate treatment can improve their quality of life.
- Setting realistic goals: Helping the patient set achievable goals related to their treatment and well-being. These goals can be small, such as rigorously following the treatment regimen for a given week, or larger, such as participating in a specific physical activity. Celebrating achievements reinforces motivation and a sense of success.
- Emotional support: Actively listening to the patient and providing emotional support. Fostering a supportive environment where the patient feels comfortable to express their emotions and fears. Seeking additional support from patient groups, therapists or counsellors, if needed.
- Teaching self-care skills: Helping the patient develop hemophilia-related self-care skills. This may include learning how to administer clotting factor infusions or recognizing the early signs of bleeding. By enabling them to take care of themselves, it will increase their sense of control and empowerment, which can be motivating.
- Celebrate successes and progress: Acknowledging and celebrating the patient’s efforts and achievements. Even small advances deserve recognition and praise. This reinforces a positive attitude and boosts motivation.
- Connecting with other patients: Helping the patient connect with other individuals who also have hemophilia. This can be through online support groups, community events or hemophilia patient camps. Sharing experiences with others facing similar challenges can be motivating and provide a sense of belonging.

It should be noted that each patient is unique, so it is important to tailor these strategies to individual needs and preferences. In addition, it is always advisable to work closely with the patient’s healthcare team to provide the best possible support [43].

The main limitations of this study are to be found in the sample itself. It is true that it is a limited sample and, in any case, subjective. The opinion of the interviewees serves to contextualize the subject treated, but it cannot be extrapolated to a more general dimension. Surely, if the study were carried out at another time and interviewing other patients, the results would not be identical. It is true that there are articles currently published with larger cohorts to address some aspect of this pathology. To counteract this limitation, it was decided to work with a specific sample size that shared a series of characteristics as described in the methodology section, as well as belonging to the same national territory, so that the information obtained would be significant and the experiences and perceptions could be compared in terms of the healthcare model, clinical management and relationship with patient associations. Another limitation of this study is the scarcity of published literature related to the subject.

As new lines of future research are derived from this study, we could highlight the application of this same interview to different hemophilia patients and family members who live in and are cared for by different health systems, in order to compare the results and obtain more evident conclusions. Extrapolating the data obtained with patients and relatives in Salamanca and being able to compare them with other regions of Spain and, of course, with other countries, will be the next objective of this line of research.

## 5. Conclusions

The main conclusions of this study are that hemophilia has a negative impact on the daily development of patients, family members and caregivers who suffer from hemophilia. The feeling of uncertainty about the future state of health and the lack of knowledge of the disease on the part of professionals causes fear, anxiety and stress. The associative movement plays a crucial role in coping with the disease showing the satisfaction of the interviewees, unlike the feeling regarding access to specific services of health institutions.

## Figures and Tables

**Table 1:** Quantitative analysis of the sample.

Total number of respondents	34
**Gender**	
Male	15
Female	19
**Respondents with hemophilia**	
Yes (patient)	26
No (family member)	8
**Age**	
20–30	4
30–40	6
40–50	16
50–60	6
60–70	1
Older 80	1
**Number of affected persons in the family**	
One	12
Two	10
Three	7
Four	1
Five	1
Six	2
Seven	1

## Data Availability

Not applicable.
